# An assessment of financial catastrophe and impoverishment from out-of-pocket health care payments in Swaziland

**DOI:** 10.1080/16549716.2018.1428473

**Published:** 2018-01-31

**Authors:** Cebisile Ngcamphalala, John E. Ataguba

**Affiliations:** ^a^ Health Economics Unit, School of Public Health & Family Medicine, University of Cape Town, Observatory, South Africa

**Keywords:** Universal health coverage, health financing, catastrophic health care payments, poverty, Swaziland

## Abstract

**Background**: As the drive towards universal coverage is gaining momentum globally, the need for assessing levels of financial health protection in countries, particularity the developing world, has increasingly become important. In Swaziland, the level of financial health protection is not clearly understood.

**Objective**: To assess financial catastrophe and impoverishment from out-of-pocket payments for health services in Swaziland.

**Methods**: The nationally representative Swaziland Household Income and Expenditure Survey (2009/2010) dataset is used for the analyses. Data are collected by the Central Statistics Office in Swaziland. The final dataset contains information on 3,167 households (i.e. about 14,145 individuals) out of the anticipated 3,750 households. Financial catastrophe is assessed using an initial threshold that is adjusted to increase with household income (i.e. rank-dependent). Payment for health services is considered catastrophic when they exceed the threshold. Impoverishment is assessed using a national poverty line and an international poverty line ($1.25/day).

**Results**: Using an initial threshold of 10.0% of household expenditure, 9.7% of Swazi households experience financial catastrophe while the proportion is estimated at 2.7% using an initial threshold of 40.0% of non-food expenditure. Between 1.0% and 1.6% of the Swazi population, representing between 10,000 and 16,000 people are pushed below the poverty line because of out-of-pocket payments. These findings indicate that financial health protection is not adequate in Swaziland.

**Conclusion**: If Swaziland is to move towards achieving universal health coverage, there is a need to address the burden created by direct out-of-pocket payments. Among other things, this means that the country needs to consider financing mechanisms that guarantee equitable access to needed quality health services, which do not place undue hardship on the poor and vulnerable.

## Background

Financial health protection is a priority area for many developing countries, especially in the move towards Universal Health Coverage (UHC) [1]. To a large extent, a country’s health financing system determines if people can use needed health services with guaranteed financial health protection. This occurs through a substantial reduction in exposure to direct out-of-pocket payments for health services [1,2].

Some of the negative consequences of out-of-pocket payments for health services include impoverishment, financial catastrophe and forgoing needed health services. In fact, a health system’s performance can be assessed against these. Briefly, impoverishment resulting from such payments arises when non-poor households are pushed into poverty solely by paying out-of-pocket for health services. This may also mean that already poor households are pushed further into poverty. Financial catastrophe, on the other hand, arises when such out-of-pocket payments exceed a certain fraction of a household’s income. This fraction is assumed as the maximum share that direct health costs can account for without disrupting the household’s standard of living, including forgoing spending on basic necessities like food [3]. Some households even incur debts or sell productive assets to cope with out-of-pocket payments [4,5]. Most often, these coping strategies are insufficient to protect the disruption of households’ welfare [6].

### Overview of health service delivery and financing in Swaziland

Health services in Swaziland are delivered at many levels – primary health care facilities, regional and national referral hospitals, and community-based care [7]. However, the country lacks a functioning referral system that leads to congestions and inefficiencies within the public health sector [8]. Overall, the public sector accounts for 45% of all health facilities while mission and private providers account for 15% and 40%, respectively [7]. Health services at the primary health care level are provided at a comparably cheaper fee than at the other levels. However, that may not necessarily imply affordability.

Total health expenditure in Swaziland increased from 5.3% of the country’s GDP in 2000 to 8.1% in 2012. This figure is very high in comparison to the average for sub-Saharan Africa (5.6% of GDP). Government expenditure on health accounts for over 18% of total government expenditure [9]. This means that Swaziland is among a few countries in sub-Saharan Africa that have met the Abuja target of devoting at least 15% of total government budgets to the health sector [10,11]. However, external resources for health remain high as they account for over one-fifth of the country’s total health expenditure [9]. Data indicate that out-of-pocket payments are minimal in Swaziland as they account for 11.5% of total health expenditure in 2012 [9]. This share is lower in Swaziland than in many other low-income countries, mostly in Africa [9,12]. In fact, keeping this share below 20% increases the potentials for financial protection [13,14]. While Swaziland records moderate shares of out-of-pocket spending in total health spending, the Annual Report of the Ministry of Health for the year 2013–2014 highlighted domestic resources mobilization for health financing as one of the country’s challenges [15]. This raises potential concern for financial health protection in Swaziland, particularly because there are cases where countries report moderate shares of out-of-pocket spending for health services but still lack adequate financial health protection [16]. Thus, it will be premature to conclude that Swaziland has adequate financial protection for health on the grounds of having a low share of out-of-pocket payments in total health financing. In fact, an understanding of the current level of financial protection will be relevant to feed into the current process of developing a National Health Financing Policy [15].

Based on the forgoing, this paper provides, for the first time in Swaziland, a comprehensive evidence of the extent and nature of financial protection in health to guide health financing policy in Swaziland. Specifically, it assesses the extent of impoverishment and financial catastrophe resulting from paying out-of-pocket for health services in Swaziland. To our knowledge, apart from an initial attempt by the World Bank [17], this assessment constitutes an initial comprehensive assessment in Swaziland. The findings will form a baseline for assessing progress towards UHC in the country.

## Methods

### Data

Data come from the Swaziland Household Income and Expenditure Survey (SHIES) 2009–2010. The SHIES is a nationally representative multi-purpose survey conducted by the Central Statistical Office of Swaziland (CSO). Households were selected based on a two-stage stratified sampling method. In the first stage, the 2007 population census framework was used to select 375 enumeration areas (EAs), with probability proportional to size. In the second stage, ten (10) households were selected within each of the 375 EAs by systematic random sampling. Thus, 3,750 households were expected to be sampled. However, the overall response rate was 84.5%. This brings the final sample size to 3,167 households with a total of 14,145 individuals [18].

### Out-of-pocket payments

Out-of-pocket payments data are obtained directly from the SHIES and included all direct costs made by households to formal and/or informal health care providers that are not reimbursed by any third party or prepayment scheme. These data are obtained from question C27 (‘how much did NAME pay for the total medical bill?’) in section C014 ‘medical expenditure (non-refundable)’ and from relevant information in the diaries that households are given for the SHIES. Briefly, the diaries are provided to all sampled households and these are filled daily to reflect daily household expenditures including out-of-pocket payments for health services. The diaries are usually blank and household members are guided to fill in items as they are purchased. These diaries have been recommended internationally to obtain more reliable estimates of household expenditures especially as they avoid the problem inherent in having a ‘long’ recall period. Specifically, out-of-pocket payments used in this paper included household spending on inpatient and outpatient services. These data have been collected in the SHIES using a 1-month recall period. However, for the purpose of reporting annual effects of out-of-pocket payments, the expenditure data are annualized (i.e. by multiplying them by 12).

### A measure of income

Total household expenditure is used as a proxy for income or living standards. This includes expenditure on frequently and infrequently purchased goods and services. These data, expressed in annual terms, are collected through the weekly diaries given to households and the general household questionnaire. The data collected through general household questionnaires were based on responses to the relevant expenditure questions reported in Section C (‘Major household expenditure’) of the household questionnaire. The use of household expenditure is in keeping with other similar studies [19–21]. A copy of the questionnaire can be accessed at <http://catalog.ihsn.org/index.php/catalog/4599/download/66270>. For assessing financial catastrophe, household expenditure is divided into total expenditure and total non-food expenditure. The rationale for this is later discussed. Wherever it is relevant, especially in the assessment of impoverishment, annualized household expenditure and out-of-pocket payments are divided by an estimated adult equivalent household size (AE) to obtain per adult equivalent expenditures [22].

(1) $$AE=(n_{A}+\alpha n_{C}{)}^{\theta }\;for\;\theta \geq 0;\;0\leq \alpha \leq 1$$

where $n_{A}$ is the number of adults in the household; $n_{C}$ is the number of children, α is the cost of children (a measure of the weight accorded to children relative to adults) and θ represents economies of scale. Following Deaton and Zaidi [23], we set α=0.5 and θ=0.75. Basically, if we set α=θ=1, then AE is interpreted as total household size for each household.

### Assessing financial protection in a health system

Two widely used measures of financial protection are considered in this paper – financial catastrophe and impoverishment resulting from paying out-of-pocket for health services [3].

### Financial catastrophe from out-of-pocket health payments

Typically, financial catastrophe is assessed by looking at the share of a household’s income (*z*%) spent out-of-pocket on health services. Any household that exceeds this share incurs financial catastrophe. Earlier methodologies [3,24] use a uniform threshold for all households irrespective of their income levels. Recently, however, there is growing concern to vary this threshold by the income levels of households [19,25]. It is argued that *z*% should not be uniform for all households but should increase with household income [19]. This is because a very small fraction of income spent on health services out-of-pocket has a larger effect on the poor than on the rich. Thus, this paper accepts the discussion regarding the need for a variable threshold [19,25] and this is applied in the paper to assess financial catastrophe in Swaziland. In the current setting in Swaziland, this methodology is justified because of the high levels of poverty and income inequality in the country. With a high poverty rate, for instance, the use of a uniform threshold is unable to capture the experiences of the poor and it is unable to discriminate between poorer and richer households. This methodology is used to compute household-specific thresholds using the SHIES data and these thresholds are in turn used to assess financial catastrophe.

This paper uses a recently proposed methodology [19] for assessing financial catastrophe. This method has also been recently applied in Uganda [26]. Briefly, this is a generalized method that can be used to report results based on the uniform threshold and rank-dependent thresholds that are dependent on household income. It does this using a parameter of aversion to inequality (γ), explained later, to define rank-dependent catastrophic payment thresholds. Essentially, when γ < 1, low-income households face lower thresholds compared to their high-income counterparts. Setting γ=1 means that all households, irrespective of their income levels, face the same threshold (*z*%). The main rationale behind the method proposed in [19] is that poorer and richer households react differently to devoting the same share of their income (e.g. 10.0%) towards financing health services out-of-pocket. Unfortunately, the use of a constant threshold does not address this as it does not recognise that ‘relatively small expenditures on health can be financially disastrous for poor households’ compared to richer households [21].

Consider an initial threshold set at $Z_{cat}$. This is a threshold (or a share) of total household expenditure or non-food expenditure. Ataguba [19] defines a rank-dependent threshold, ${Z_{cat}}^{\prime }$, as:

(2) $${Z_{cat}}^{\prime }=\omega \left(p;\gamma \right)\cdot Z_{cat}$$

where $\omega \left(p;\gamma \right)=\gamma (1-p{)}^{\gamma }$, p represents a household’s percentile when households are ordered in ascending order of income and 0 < γ≤1. Except otherwise stated, this paper uses γ=0.8 to estimate the rank-dependent thresholds. Initial thresholds considered include 5%, 10%, 15%, 20% and 25% of total household expenditure and 5%, 10%, 15%, 25% and 40% of non-food expenditure.

### Financial catastrophe (headcount)

Let $y_{i}$ be total household expenditure (or non-food expenditure) for household i and $T_{i}$ be that household’s total out-of-pocket payments. We can define ${O_{i}}^{\prime }$ to represent the rank-dependent catastrophic overshoot. This represents the proportion by which household i exceeds the threshold (${Z_{cat}}^{\prime }$). In essence, ${O_{i}}^{\prime }=\left(T_{i}/y_{i}\right)-{Z_{cat}}^{\prime }$ if the household exceeds the threshold and ${O_{i}}^{\prime }=0$ if the household does not.

If ${E_{i}}^{\prime }$ is a measure that indicates whether a household exceeds the rank-dependent threshold, then ${E_{i}}^{\prime }=1$ when ${O_{i}}^{\prime }\;>\;0\;$and zero otherwise. Thus, the rank-dependent headcount [19] is estimated as:

(3) $${H_{cat}}^{\prime }=\frac{\left(\sum_{i=1}^{N}{E_{i}}^{\prime }\right)}{N}=\mu_{E^{\prime }}$$

where $\mu_{E^{\prime }}$ is the mean of ${E_{i}}^{\prime }$ and *N* is the total number of households. This measure indicates the percentage of households whose out-of-pocket payments as a fraction of their income exceed the rank-dependent threshold (${Z_{cat}}^{\prime }$).

### Financial catastrophe (gap)

The rank-dependent catastrophic gap (${G_{cat}}^{\prime }$) measures the difference between $\left(T_{i}/y_{i}\right)$ and ${Z_{cat}}^{\prime }$ for each household where ${E_{i}}^{\prime }=1$. It is computed as [19]:

(4) $${G_{cat}}^{\prime }=\frac{\left(\sum_{i=1}^{N}{O_{i}}^{\prime }\right)}{N}=\mu_{O^{\prime }}$$

where $\mu_{O^{\prime }}$ is the mean of $O^{\prime }$ across all households.

Another measure of financial catastrophe is the mean positive rank-dependent gap $\left(P{G_{cat}}^{\prime }\right)$. This is similar to the catastrophic gap but averaged across households that incur financial catastrophe. Together, they indicate the intensity of financial catastrophe. $P{G_{cat}}^{\prime }$ is computed as [19]:

(5) $$P{G_{cat}}^{\prime }=\frac{\left(\sum_{i=1}^{N}{O_{i}}^{\prime }\right)}{\left(\sum_{i=1}^{N}{E_{i}}^{\prime }\right)}=\frac{\mu_{O^{\prime }}}{\mu_{E^{\prime }}}$$

Specifically, $P{G_{cat}}^{\prime }$ measures the average share by which those that incur financial catastrophe have exceeded the threshold (${Z_{cat}}^{\prime }$).

It is worth mentioning that the indices from Equations (3–5) can be obtained using either catastrophic thresholds of total household expenditure or total household non-food expenditure.

### The impoverishing effect of out-of-pocket payments

The impoverishment effect of out-of-pocket spending is assessed by examining the extent to which individuals are pushed below the poverty line as a result of paying out-of-pocket for health services [3]. Indices of interest include the impoverishment headcount and gap (including the normalised and mean positive gap).

### Impoverishment headcount

If $z_{pov}$ is the poverty line and $x_{i\;}$is individual $i^{\prime }s$ pre-payment income (i.e., income before paying out-of-pocket for health services), then define $P_{i}^{pre}=1\;if\;x_{i\;}\;<\;z_{pov}$ and $P_{i}^{pre}=0\;$otherwise.

The pre-payment poverty headcount is estimated as [3]:

(6) $$H_{pov}^{pre}=\frac{\sum_{i=1}^{N}P_{i}^{pre}}{N}=\mu_{p^{pre}}$$

where *N* is the sample (population) size.

An analogous expression can be written for the post-payment poverty headcount by replacing prepayment income with post-payment income such that $H_{pov}^{post}=\mu_{p^{post}}$.

### Impoverishment gap

The prepayment poverty gap $\left(g_{i}^{pre}\right)$ measures the extent to which incomes are below the poverty line. These shortfalls are larger for poorer individual. Also, the poverty gap can be interpreted as the costs for eliminating poverty per head relative to the poverty line. This gap is equal to $z_{pov}-x_{i\;}$ if $x_{i\;}\;<\;z_{pov}$, and zero otherwise. The associated pre-payment poverty gap is defined as [3]:

(7) $$G_{pov}^{pre}=\frac{\sum_{i=1}^{N}g_{i}^{pre}}{N}=\mu_{g^{pre}}$$

The poverty gap in Equation (7) can be expressed as a share of the poverty line to obtain the normalised pre-payment poverty gap defined as $NG_{pov}^{pre}=G_{pov}^{pre}/z_{pov}$. This is particularly useful for making cross-country comparisons. The poverty gap can also be averaged across only impoverished individuals to obtain the mean positive pre-payment poverty gap defined as [3]:

(8) $$MPG_{pov}^{pre}=\frac{\sum_{i=1}^{N}g_{i}^{pre}}{\sum_{i=1}^{N}p_{i}^{pre}}=\frac{\mu_{g^{pre}}}{\mu_{p^{pre}}}$$

Again, using post out-of-pocket payment income and replacing the ‘pre’ superscript with ‘post’ will yield the post-payment versions of all these indices

The impoverishing impact of out-of-pocket payments is defined as the difference between the relevant pre-payment and post-payment indices.

For example, the impoverishing impact on headcount is obtained as [3]:

(9) $$PI^{H}=H_{pov}^{post}-H_{pov}^{pre}$$

The Swazi national poverty line (E461 per person/per month) and the international ($1.25/day at the 2005 purchasing Power Parity (PPP)) poverty line are used in the analysis. In 2010, $1 = E4.7 [27].

## Results

### Financial catastrophe

The results presented in Table 1 show that substantial levels of financial catastrophe exist in Swaziland. Setting the initial threshold level at 10.0% of total household expenditure, the catastrophic headcount is estimated at 9.7%. This means that, on average, financial catastrophe is experienced by 9.7% of Swazi households at this initial threshold. This translates to over 21,000 households (or >97,000 individuals) based on an estimated population of about 1 million people [18]. As expected, the catastrophic headcount ratio decreases when a higher initial threshold value is used. For example, only 2.4% of Swazi households (>5,300 households) experience financial catastrophe when the initial threshold is set at 25% of total household expenditure.

**Table 1.** Households’ catastrophic out of-pocket health expenditures indices using rank-dependent thresholds.

	Total household expenditure				Total non-food household expenditure			
Initial threshold (*z*) of household expenditure	**5%**	**10%**	**20%**	**25%**	**5%**	**10%**	**25%**	**40%**
Catastrophic headcount $\left({H_{cat}}^{\prime }\right)$	16.8%	9.7%	3.8%	2.4%	24.2%	15.8%	5.9%	2.7%
Catastrophic gap $\left({G_{cat}}^{\prime }\right)$	1.5%	0.9%	0.4%	0.3%	2.9%	2.1%	0.9%	0.4%
Catastrophic mean positive gap $\left(P{G_{cat}}^{\prime }\right)$	8.6%	9.5%	10.6%	11.7%	12.1%	13.3%	14.7%	13.3%

Notes: The parameter of aversion to inequality (γ) used to generate the indices is set at 0.8

The analysis is based on a total of 3,167 households

Overall, the results in Table 1 show that catastrophic headcount varied with the initial threshold as well as the measure for households’ expenditure. For example, defining financial catastrophe using total household expenditure and non-food expenditures at the 5% initial threshold, an estimated 16.8% (>37,000 households) and 24.2% (>54,000 households) of Swazi households incurred catastrophic payments, respectively. A similar pattern is observed with respect to the gap measures for both expenditure measures. For total expenditure at the 5.0% and 10.0% initial thresholds, households paid out-of-pocket health payments in excess (overshoot) of 1.4% and 0.9%, respectively of their rank-dependent thresholds.

Of importance is the mean positive catastrophic gap. For total household expenditure, as shown in Table 1, the mean positive gap ranges from 8.6% (at the 5% initial threshold) to 11.7% (at the 25% initial threshold). For total non-food expenditure, these gaps are larger, ranging from 12.1% to 14.7%.

### Impoverishment

Using the Swaziland national poverty line, as shown in Table 2, poverty headcount increased from 62.3% to 63.3% due to out-of-pocket payments. This translates to 1.0% of the Swazi population impoverished by paying out-of-pocket for health services. This represents over 10,000 individuals pushed below the national poverty line. Relatively, poverty headcount in Swaziland increased by 1.6% because of households’ out-of-pocket payments. The poverty gap increased by E56.90 or a 3.4% relative increment due to out-of-pocket payments.

**Table 2.** Poverty headcounts and gap measures.

National poverty line (E461 per person per month)					International poverty line ($1.25/day)			
	Prepayment (a1)	Post-payment (b1)	Absolute difference (c1) = (b1)-(a1)	Relative difference (d1) = (c1)/(a1)	Prepayment (a2)	Post-payment (b2)	Absolute difference (c2) = (b2)-(a2)	Relative difference (d2) = (c2)/(a2)
Poverty headcount	62.3%	63.3%	1.0%	1.6%	20.9%	22.5%	1.6%	7.7%
Poverty gap	1662.2	1719.1	56.9	3.4%	145.1	155.9	10.8	7.4%
Normalized gap	30.0%	31.0%	1.0%	3.3%	6.7%	7.2%	0.5%	7.5%
Normalised mean positive gap	48.3%	49.1%	0.8%	1.7%	32.2%	32.0%	−0.2%	−0.6%

Note: Total number of households = 3,167.

When the poverty gap is expressed as a proportion of the national poverty line, the resulting normalised poverty gap increased by 1.0% and 3.3% in absolute and relative terms, respectively. Also, when the normalised poverty gap is assessed only among the poor, it increased by 0.8% representing a relative increment of 1.7%. This means that among the poor, on average, poverty is deepened further by 0.8% because of out-of-pocket health payments.

A similar pattern of increased numbers of individuals pushed below the poverty line due to out-of-pocket payments is evident using the international poverty line. An interesting result from using the international poverty line relates to the normalised mean positive gap. This gap decreased when the international poverty line is used compared to an increment reported using the national poverty line. This is attributed mainly to more individuals being pushed below the international poverty line and not because the poverty of the already poor is deepened.

## Discussion

This paper sets out with the aim of assessing financial health protection in Swaziland using two broad measures – financial catastrophe and impoverishment from paying out-of-pocket for health services. Briefly, this paper indicates that a considerable percentage of households experienced catastrophic health expenditures at all the threshold levels of total household expenditure and non-food expenditure. However, as expected, the severity significantly decreases at higher threshold levels. These results suggest inadequate financial health protection in Swaziland’s health systems, even though the country records a modest share (<15.0%) of out-of-pocket health payments in total health expenditure that is considered to be a relatively small percentage [13]. Based on previous studies, this lack of financial health protection in Swaziland may suggest that the burden is heavier among poorer households and this has implications for their consumption of essential non-medical necessities. This is the case as poorer households, compared to richer ones, allocate a greater share of their budget to food [21,28].

This paper estimated that 9.7% of Swazi households incur financial catastrophe using an initial threshold of 10.0% of total household expenditure. This proportion dropped to 2.7% of Swazi households using an initial threshold of 40.0% of household non-food expenditure. The initial assessment of financial catastrophe by the World Bank using the 2003 World Health Survey and a uniform threshold indicates that 18.5% of Swazi households spend more than 10.0% of their consumption out-of-pocket on health services. This proportion decreased to 9.2% at the 40.0% threshold of total consumption [17]. While the pattern is similar, direct comparison of results is difficult as there are disparities between these two studies conducted in Swaziland. The disparities are mainly due to differences in data and methodology of assessment. Out-of-pocket spending obtained from the World Health Survey is aggregated at the point of collection compared to the more detailed diary method used to collect same data, but disaggregated, in the SHIES. Also, unlike the rank-dependent thresholds used in this paper [19], the World Bank’s study used a uniform threshold for assessing financial catastrophe. However, our results are similar in pattern to those reported for other developing countries. In Uganda, using rank-dependent thresholds, at the 5% and 25% initial threshold levels of total household expenditure, the incidence of catastrophic health expenditure stood at 38.0% and 6.7%, respectively [26]. In Rwanda at the 10.0% and 40.0% uniform threshold levels of household non-food expenditure, the incidence of catastrophe was estimated at 16.2% and 2.9%, respectively [29]. In Ghana, data from 2005/06 show that between 2.4% and 11.0% of households incur financial catastrophe, depending on the choice of threshold levels of total household and non-food expenditures [30]. In Kenya using the 40.0% uniform threshold level of household non-food expenditure, 6.6% of Kenyan households incurred financial catastrophe in 2013 [31].

The other set of analyses in this paper estimated that 1.0% of the Swazi population (i.e. over 10,000 Swazis) are pushed below the poverty line due to out-of-pocket health payments. The proportion is even higher (1.6%) when the international poverty line is used. The findings are comparable to those of other low-income countries in Africa [32,33]. Although this percentage is small, it represents a significant population given the high national poverty rate estimated at 63.0% [18]. Furthermore, using the national poverty line, the normalised mean positive poverty gap increased by 1.7% (relatively). This means that the increase in poverty caused by out-of-pocket payments is mainly because of the deepening of poverty for the previously poor households. Similar results have been observed in earlier studies conducted in other developing countries. In Ghana, for instance, using the international $1.25/day poverty line, 1.6% of Ghanaians were pushed into poverty by paying out-of-pocket for health services. This translated into a 2.3% increment in the normalized poverty gap, indicating deepening of poverty among the poor [34].

While our results are similar in pattern to those of other studies, they may understate the extent of financial catastrophe and impoverishment in Swaziland. This is because of the high national poverty rate and the fact that the country is still placing charges on the use of health care services even at public facilities. Based on literature from elsewhere, in this circumstance, the poor may be under-utilising health care services, as they may not afford payment. They may also modify their perception of illness [35]. This has more devastating consequences on households’ welfare [6]. A similar finding also emerged in a cross-country study that included Swaziland [4]. However, it is important to note that the study was assessing households’ coping mechanisms and not financial catastrophe or impoverishment *per se*.

The lack of financial protection in the Swaziland health system is a cause for concern. There is therefore an urgent need for the country to institute financial-risk protection measures to protect households, particularly the poor, from financial risks. An example is the use of mandatory prepayment mechanisms that have been recommended by the World Health Organization [36] and which have been demonstrated as effective in bringing down the levels of financial risk even in low income countries [14,29,37].

The study findings do not only add to the growing body of literature, but they also enhance better understanding of the financial health protection status in Swaziland. They serve as a baseline for future-related studies to be conducted in the country, especially using the recently concluded SHIES 2016/17 that is yet to be available in the public domain. In addition, policy makers can use the findings demonstrated in this study for interventions targeting those households at risk. Additionally, the study findings could serve as an advocacy tool to politicians for the need to address the lack of financial protection. Further, it has the potential to place financial health protection on top of the Ministry of Health’s agenda as it has demonstrated critical, relevant and limited evidence in the country. A key strength of the study is that financial catastrophe is assessed using variable thresholds other than the conventional uniform threshold used by most previous studies. This methodology has the advantage of explicitly recognising the diminishing marginal utility of income and it incorporates the principle of vertical equity, which is fundamental in the assessment of fairness in health financing system. Importantly, studies using the variable threshold approach have reported higher estimates compared to those obtained with the uniformed threshold [19,26]; signifying an understatement of financial catastrophe.

The paper has a few limitations. One of the limitations lies in the fact that the methodologies for assessing financial catastrophe and impoverishment from out-of-pocket health care payments do not consider those who need services but could not afford. Evidence suggests greater welfare loss among these households compared to those that are captured as incurring catastrophic payments [38]. Thus, the estimates reported in this paper could be underestimated. Another limitation is the use of an arbitrary initial threshold (*z*%) for the assessment of financial catastrophe. Unfortunately, no threshold is universally accepted for such assessment. However, based on international recommendations [3,28], the results of financial catastrophe have been presented using a range of initial thresholds to assess the impact of choosing thresholds on the overall pattern of financial catastrophe. These results indicate the expected patterns. Relatedly, the methodology used in this paper for assessing financial catastrophe computes different thresholds for different households depending on their expenditure levels. While this is intuitive, it remains a debate as to how different the thresholds should be. This remains an area for future research especially in the context of vertical equity in health financing. Also, the 2009/10 data used for analysis may be relatively old for timely policy making. However, this represents the most recent SHIES dataset that is available to the public. The potential public release of the 2016/17 SHIES data will provide an opportunity to update the analyses and the results in this article will serve as baseline information. Similarly, the potential limitations inherent in the use of cross-sectional survey data such as recall bias cannot be disregarded. However, our results reported are qualitatively similar to those reported in related studies.

This paper’s findings point to the need for future research to explore factors that increase households’ vulnerability to financial risks. This should go beyond individual- or household-level characteristics. It would also be important to explore, within the context of Swaziland, the possible reasons why households use different types of health facilities and how that impact on financial-risk protection to better inform interventions in future.

## Conclusion

The study findings demonstrated that financial health protection is inadequate in Swaziland even though out-of-pocket payments comprise a small share (<15.0%) of total health finance in the country. It emerged that between 2.4% and 24.2% of Swazi households incur financial catastrophe depending on the choice of initial catastrophic threshold. Also, paying out-of-pocket for health care impoverishes between 1.0% and 1.6% of the Swazi population. If Swaziland is to move towards achieving UHC, there is a need for the country to devise a strategy to protect and prevent such households from financial risk. Importantly, based on international literature, the country should rely more on health financing mechanisms that can be pooled equitably and that do not impose undue hardship on the poor and vulnerable. This could involve the establishment of a National Health Insurance or providing free health services for those that cannot afford any payment.

## Paper context

Financial-risk protection is an important issue within developing countries. Countries have been encouraged to ensure adequate financial health protection for their population. However, there is a dearth of any comprehensive study in Swaziland showing the extent of such protection. This paper shows that a substantial number of Swazi population lacks financial health protection. These findings are relevant for the current process of developing a detailed health financing strategy in Swaziland.
